# Food Addiction and Grazing—The Role of Difficulties in Emotion Regulation and Negative Urgency in University Students

**DOI:** 10.3390/nu15204410

**Published:** 2023-10-17

**Authors:** Andreia Ribeiro, Jorge Sinval, Sílvia Félix, Carolina Guimarães, Bárbara César Machado, Sónia Gonçalves, Marta de Lourdes, Eva M. Conceição

**Affiliations:** 1 Psychology Research Center (CIPsi), School of Psychology, University of Minho, 4710-057 Braga, Portugal; a81349@uminho.pt (A.R.); sia_felix@hotmail.com (S.F.); carolinaogmrs@gmail.com (C.G.); sgoncalves@psi.uminho.pt (S.G.); martamagalhaeslourdes@outlook.com (M.d.L.); 2 National Institute of Education, Nanyang Technological University, Singapore 637616, Singapore; jorge.sinval@nie.edu.sg; 3 Department of Evidence-Based Health, Escola Paulista de Medicina, Universidade Federal de São Paulo, São Paulo 04023-062, SP, Brazil; 4 Faculty of Philosophy, Sciences and Languages of Ribeirão Preto, University of São Paulo, Ribeirão Preto 14040-901, SP, Brazil; 5 Business Research Unit (BRU-IUL), Instituto Universitário de Lisboa (ISCTE-IUL), 1649-026 Lisbon, Portugal; 6 Research Centre for Human Development (CEDH), Faculty of Education and Psychology, Universidade Católica Portuguesa, 4169-005 Porto, Portugal; bcmachado@ucp.pt; 7 Faculty of Psychology and Educational Sciences, University of Porto, 4200-135 Porto, Portugal

**Keywords:** food addiction, grazing, emotional regulation, negative urgency, university students

## Abstract

University students are a vulnerable population to the development of disordered eating, such as food addiction (FA) and grazing. FA is an emerging concept characterized by an intense desire to eat hyper-palatable foods. Grazing is characterized by the repetitive and unplanned ingestion of food throughout a period of time. Both FA and grazing have been associated with increased scores of negative urgency (NU) and difficulties in emotion regulation (ER). This study aims to evaluate the frequency of FA and grazing in a university population and to test the direct, total, and indirect effects—via FA—of ER and NU on repetitive eating and compulsive grazing. A total of 338 participants responded to a set of psychological measures assessing these variables. Thirty-six (10.7%) participants met the criteria for FA diagnosis and 184 (54.4%) presented grazing. Confirmatory factor analysis showed acceptable fit indexes for the model tested (χ^2^_(1695)_ = 3167.575; *p* < 0.001; *CFI* = 0.955; *NFI* = 0.908; *TLI* = 0.953; *SRMR* = 0.085; *RMSEA* = 0.051; *CI* 90% (0.048; 0.053); *P*[*RMSEA* ≤ 0.05] = 0.318) and suggested that FA partially mediated the effect of difficulties in ER and NU on grazing, specifically on compulsive grazing. The results indicate that individuals with difficulties in ER and impulse control under negative emotions are more likely to engage in grazing if food addiction scores are higher. These results highlight the importance of assessing these variables, particularly in at-risk populations such as university students.

## 1. Introduction

The transition to university is considered a critical period for the development of eating disorders, as it often involves changes in students’ eating habits. This life stage is generally associated with changes in the family environment, academic stress, and new responsibilities [1,2], which can lead to an increase in skipping meals and in the consumption of snacks, alcohol, unhealthy food choices, and a tendency towards higher-caloric options (e.g., fast food) [3,4,5]. Hence, college students have been conserved as a high-risk population for the development of problematic eating behaviors [6].

Accordingly, the literature shows that university students exhibit more food addiction than the general population [7]. Although the concept of food addiction is not yet widely accepted, it has been extensively studied in the last decade, being conceptualized by a strong urge to overeat hyper-palatable foods [8,9]. Ultra-processed foods are high in salt, fat, sugar, and preservatives, making them highly pleasurable. The underlying mechanisms leading to increasing cravings for these foods resemble the pattern of symptoms seen in substance use disorders (e.g., alcohol), and some authors have suggested a potential for addiction [10,11,12,13]. Consuming hyper-palatable foods can activate the brain’s reward system, releasing neurotransmitters such as dopamine, which enhance feelings of pleasure and reinforcement. Repeated activation can lead to dependence, as individuals feel compelled to consume larger quantities of these hyper-palatable foods to avoid negative withdrawal symptoms (both physical and emotional) [11,14,15].

Food addiction has often been associated with problematic eating behaviors [7], such as grazing behavior [12,16]. For example, Bonder et al. (2018) [12] demonstrated a positive relationship between compulsive grazing and the severity of food addiction symptoms in university students. Grazing is characterized by the repetitive consumption of small/modest amounts of food in a short period of time or throughout the day, in an unplanned manner and not in response to hunger cues. In addition to the repetitive nature of grazing, on some occasions, a compulsive nature is also present, resulting in an eating pattern associated with increased psychopathology (compulsive grazing) [17,18,19].

Both grazing and food addiction have been linked to psychological variables, such as difficulties in emotional regulation and negative urgency [20]. Studies have shown that emotion regulation difficulties were predictors of food addiction symptoms [21], and individuals diagnosed with food addiction had more difficulties in emotional regulation [20,22,23]. Emotional regulation involves resorting to adaptive strategies to deal more appropriately with emotions without suppressing them and, ultimately, controlling one’s behavioral emotion regulation compasses the following aspects: awareness, understanding, and acceptance of one’s own emotions, the ability to execute goal-oriented behaviors, controlling impulses when experiencing negative emotions, and perceiving that such emotional regulation strategies are effective [24]. The absence of these skills indicates the existence of difficulties in emotional regulation and may contribute to increased food consumption as a maladaptive strategy to cope with emotions [20,25].

In this regard, negative urgency has been associated with food addiction [20,26,27,28] and grazing behaviors [19] and refers to the tendency to take impulsive actions in response to negative emotional states [26]. Thus, in the presence of intense emotional states, individuals with higher levels of impulsivity make quicker decisions regarding food cues, engaging in addictive and problematic eating behaviors to avoid feelings such as anxiety or physical withdrawal symptoms.

**Figure 1:**
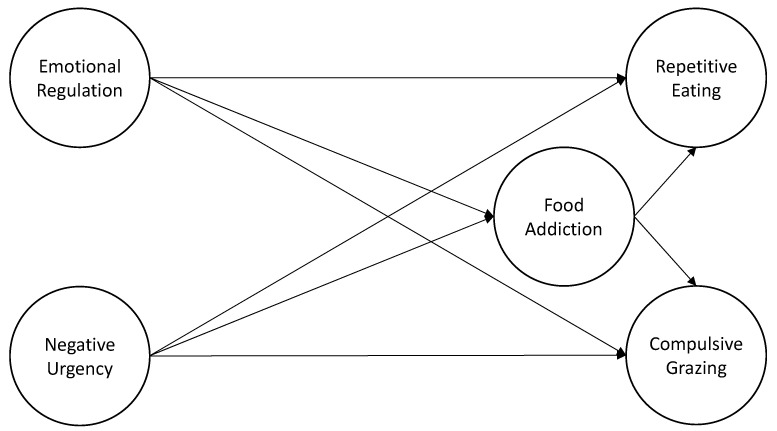
Hypothesized model associations between emotional regulation and negative urgency with food addiction and grazing (both subscales: Repetitive eating and Compulsive grazing).

According to this literature, negative urgency and difficulties in emotional regulation appear to be highly associated with food addiction and grazing. However, this association has not been well explored in community populations. Considering the existing literature, we hypothesized a model in which food addiction serves as a mediating variable between the effect of difficulties in emotional regulation, negative urgency, and grazing (compulsive grazing and repetitive eating) (model depicted in Figure 1). A deeper understanding of these factors and related variables provides further insights into the role of food addiction in understanding grazing behavior as a maladaptive emotion modulation strategy in at-risk populations.

This study has two main objectives: (1) to assess and characterize the frequency of food addiction and grazing in a university population, and (2) to test the direct, total, and indirect effects—via food addiction—on repetitive eating and compulsive grazing (Figure 1).

## 2. Materials and Methods

### 2.1. Participants and Procedure

The participants recruited for this cross-sectional study were students from a Portuguese University. Inclusion criteria were being over 18 years old and understanding the written and spoken Portuguese language.

Students attending for a Psychology degree were invited to participate using the Accreditation System for Participation in Experiences implemented by the School of Psychology. Other students were invited through an email sent via the institutional mailing list with information about the study and a link to access an online questionnaire on Google Forms. The participants accepted the informed consent document that explained the goals, procedures, and voluntary nature of the study. Subsequently, they responded to a set of self-reported questionnaires.

### 2.2. Measures

#### 2.2.1. Sociodemographic and Clinical Questionnaire

This questionnaire was created for the present study to collect sociodemographic characteristics: age, sex, nationality, marital status, level of education in attendance, specific course in attendance, and professional status. Also, a few other questions assessed anthropometric and clinical information, such as height, weight, weight satisfaction, and previous history of eating disorders.

#### 2.2.2. Portuguese Yale Food Addiction Scale 2.0 (P-YFAS 2.0; [29,30])

This is a self-reported measure composed of 35 items scored on an 8-point ordinal scale (0—“Never”; 7—“Every day”) that assesses symptoms of food addiction in the last 12 months. The items were developed based on the DSM-5 criteria for substance use disorder and adapted to assess the following 11 symptoms of food addiction (plus clinical impairment): (1) overeating; (2) desire to cut down; (3) time spent eating/searching for food; (4) food craving; (5–7) related impairment (work/school, family, social relationships); (8–9) risky use (detrimental physical/psychological consequences); (10) tolerance to food; and (11) withdrawal. According to this measure, a diagnosis of food addiction required scoring on two or more of the symptoms assessed in combination with significant clinical impairment [31]. A final symptom count score was calculated to inform about food addiction severity by adding up all symptoms (Mild: 2–3 symptoms; Moderate: 4–5 symptoms; and Severe: 6 symptoms).

#### 2.2.3. Repetitive Eating Questionnaire (Rep(eat)-Q; [18])

The Repetitive Eating Questionnaire is a 12-item questionnaire that assesses grazing eating behavior. The items are responded to through a 7-point ordinal scale (0—“never”, 6—“every day”), which results in a total score and two subscales: compulsive grazing and repetitive eating. The cutoff point for the presence of grazing, established for this population, was 1.25 (for the total score), and higher scores indicate a more pronounced grazing eating pattern [18].

#### 2.2.4. Difficulties in Emotion Regulation Scale–Short Form (DERS-SF; [24,32,33])

The Difficulties in Emotion Regulation Scale–Short Form is an 18-item measure that assesses global difficulties in emotion regulation and six specific emotion regulation domains: limited access to emotion regulation strategies, non-acceptance of emotional responses, lack of emotional awareness, difficulties in impulse control, difficulties in implementing goal-directed behaviors, and lack of emotional clarity. Higher scores indicate higher difficulties in emotion regulation.

#### 2.2.5. UPPS-P–Negative Urgency Subscale (UPPS-P; [34,35])

This UPPS-P–Negative Urgency Subscale assesses the tendency to act impulsively in response to intense negative emotions throughout 12 items assessed using a Likert scale, from 1—“completely agree” to 5—“completely disagree”. Higher scores are indicators of more negative urgency.

### 2.3. Statistical Analysis

The statistical analyses were performed using IBM^®^ SPSS^®^ Statistics version 28 and the *R* statistical programming language [36] via the integrated development environment, *RStudio* [37].

Measures of central tendency and dispersion were completed for sample characterization purposes. Then, Pearson correlation coefficients were used to investigate associations between the variables under study.

Structural equation modeling (SEM) tools were used to test the mediation model (depicted in Figure 1) in which food addiction would be the mediating variable of the relationship between difficulties in emotion regulation and negative urgency in the presence of grazing. The full SEM model (i.e., with latent regressions) was estimated using the *lavaan* package version 0.6-16 [38]. A significance level of 5% was used for all analyses (α = 0.05). As goodness-of-fit indices, the χ^2^ statistic, the *CFI* (Comparative Fit Index), the *NFI* (Normed Fit Index), the *TLI* (Tucker–Lewis Index), the *SRMR* (Standardized Root Mean Square Residual), and the *RMSEA* (Root Mean Square Error of Approximation) were used to assess the model fit. The scaled versions of the goodness-of-fit indices were used, except for SRMR, which does not have a scaled version. Estimates of *CFI*, *NFI*, and *TLI* above 0.95 and of *SRMR* and *RMSEA* below 0.08 were considered satisfactory [39,40].

The dimensionality of the psychometric instruments was assessed using the Confirmatory Factor Analysis (CFA) technique [41] with the Weighted Least Squares Means and Variances (WLSMV) estimator [42]. The choice for the WLSMV estimator was justified due to the absence of the requirement for multivariate normality as an assumption and the ordinal nature of the indicators in all psychometric instruments. The CFA was estimated using the *lavaan* package [38]. Modifications indices (MI) above 11 (*p* < 0.001) were inspected in the light of the theory [43].

The Average Variance Extracted (*AVE*) [44] and the ω estimators were used to assess the evidence of the reliability of the scores in terms of internal consistency for the first-order latent factors. Satisfactory values were considered to be *AVE* ≥ 0.5 and ω ≥ 0.8. Three estimators of internal consistency were used for the second-order latent factors: the fraction of the variability of a composite score calculated from the observed indicators that were attributable to the second-order factor (ω*_L_*_1_), the fraction of the variability among first-order common factors that were attributable to the second-order factor (ω*_L_*_2_), and the fraction of the observed variability explained by the second-order factor after partialing out the uniqueness from the first-order factors (ω*_partial L_*_1_). The *semTools* package version 0.5-6.925 [45] was used to obtain the estimates for all estimators of internal consistency (both first and second-order.

## 3. Results

### 3.1. Sample Characterization

The sample of this study included 338 university students from the University of Minho (91.4% females), aged between 18 and 35 years (*M* = 20.98, *SD* = 3.24). The mean self-reported BMI was 22.25 (*SD* = 3.33).

One hundred and eighty-four (54.4%) participants presented grazing behavior, and thirty-six (10.7%) fulfilled the criteria for a diagnosis of food addiction according to YFAS 2.0 (i.e., the presence of two or more symptoms of food addiction accompanied by significant clinical impairments/distress). Of the total of participants with food addiction, thirty-one (86.1%) were female, ten participants (27.8%) scored within the mild range of food addiction, five (13.9%) in the moderate range, and twenty-one (58.3%) in the severe range. As for the symptomatology of food addiction, the results indicate that the most frequent diagnostic criteria (except for clinical impairment) were withdrawal symptoms (77.8%), the persistence of consumption despite its negative physical or emotional consequences (75%), and the continuous desire or inability to reduce/stop consumption (69.4%).

### 3.2. Association between Food Addiction Symptomatology, Grazing, and the Variables under Study

The Pearson′s correlation coefficients test revealed statistically significant positive correlations between the food addiction scores and the compulsive grazing subscale, repetitive eating subscale, difficulties in emotional regulation, and negative urgency. Both compulsive grazing and repetitive eating showed statistically significant positive correlations with difficulties in emotional regulation and negative urgency. These results are summarized in Table 1.

### 3.3. Measurement Model

#### 3.3.1. Dimensionality

All instruments were individually analyzed in terms of their dimensionality—via CFA—and reliability of the scores—in terms of internal consistency. The YFAS’ initially presented an unsatisfactory fit to the data (χ^2^_(560)_ = 2126.059; *p* < 0.001; *CFI* = 0.941; *NFI* = 0.921; *TLI* = 0.937; *SRMR* = 0.114; *RMSEA* = 0.091; 90% *CI* (0.087; 0.095); *P*[*RMSEA* ≤ 0.05] < 0.001). After analyzing the MI, five correlations (i.e., MI > 100) among the items’ residuals were added (*r_item_*_1,_*_item_*_2_ = 0.51; *r_item_*_12,_*_item_*_13_ = 0.68; *r_item_*_16, *item* 17_ = 0.70; *r_item_*_29,_*_item_*_30_ = 0.61; *r_item_*_31,_*_item_*_32_ = 0.84); all of them were statistically significant (*p_i_* < 0.001). The resulting fit of the modified model to the data was acceptable (χ^2^_(555)_ = 1584.092; *p* < 0.001; *CFI* = 0.961; *NFI* = 0.941; *TLI* = 0.958; *SRMR* = 0.100; *RMSEA* = 0.074; 90% *CI* (0.070; 0.078); *P*[*RMSEA* ≤ 0.05] < 0.001).

The Rep(eat)-Q original model presented an unsatisfactory fit to the data (χ^2^_(53)_ = 228.204; *p* < 0.001; *CFI* = 0.981; *NFI* = 0.976; *TLI* = 0.976; *SRMR* = 0.038; *RMSEA* = 0.103; 90% *CI* (0.090; 0.117); *P*[*RMSEA* ≤ 0.05] < 0.001). The MI was inspected (i.e., MI > 20); three statistically significant (*p_i_* < 0.001) pairs of items’ residuals correlations were added (*r_item_*_2,_*_item_*_3_ = 0.37; *r_item_*_2,_*_item_*_4_ = 0.48; *r_item_*_3,_*_item_*_4_ = 0.42). The modified model presented an acceptable fit to the data (χ^2^_(50)_ = 228.204; *p* < 0.001; *CFI* = 0.981; *NFI* = 0.976; *TLI* = 0.976; *SRMR* = 0.038; *RMSEA* = 0.103; 90% *CI* (0.090; 0.117); *P*[*RMSEA* ≤ 0.05] < 0.001).

The original DERS-SF model presented a poor fit to the data (χ^2^_(135)_ = 2221.259; *p* < 0.001; *CFI* = 0.850; *NFI* = 0.842; *TLI* = 0.830; *SRMR* = 0.158; *RMSEA* = 0.214; 90% *CI* (0.206; 0.222); *P*[*RMSEA* ≤ 0.05] < 0.001). After inspecting the model and the corresponding MI, nine items were removed, one due to its low factor loading (λ*_item_*_6_ = 0.283) and eight due to the MI values (items 1, 2, 3, 4, 8, 9, 11, 12, 17). The modified DERS-SF model presented a satisfactory fit to the data (χ^2^_(20)_ = 82.201; *p* < 0.001; *CFI* = 0.974; *NFI* = 0.966; *TLI* = 0.964; *SRMR* = 0.046; *RMSEA* = 0.096; 90% *CI* (0.075; 0.118); *P*[*RMSEA* ≤ 0.05] < 0.001).

The UPPS-NU original model did not present an acceptable fit to the data (χ^2^_(54)_ = 593.505; *p* < 0.001; *CFI* = 0.907; *NFI* = 0.899; *TLI* = 0.886; *SRMR* = 0.089; *RMSEA* = 0.172; 90% *CI* (0.160; 0.185); *P*[*RMSEA* ≤ 0.05] < 0.001). Item 11 (the only item that was not reversely scored) was removed due to its low factor loading (λ = 0.415). Six items were removed after inspection of the MI (i.e., Items 1, 2, 3, 4, 5, 10). The resulting modified version presented a perfect fit to the data (χ^2^_(5)_ = 3.898; *p* = 0.564; *CFI* = 1.000; *NFI* = 0.999; *TLI* = 1.001; *SRMR* = 0.009; *RMSEA* = 0.000; 90% *CI* (0.000; 0.067); *P*[*RMSEA* ≤ 0.05] = 0.865).

#### 3.3.2. Reliability: Internal Consistency

In terms of reliability of the scores, the estimates in terms of internal consistency presented satisfactory to very satisfactory values for the YFAS’ dimension (ω = 0.96; *AVE* = 0.63), for the DERS’ dimension (ω = 0.87; *AVE* = 0.53) and for the UPPS-NU subscale (ω = 0.89; *AVE* = 0.70). The Rep(eat)-Q first-order dimensions also presented very satisfactory estimates for compulsive grazing (ω = 0.92; *AVE* = 0.73) and repetitive eating (ω = 0.88; *AVE* = 0.69). The second-order dimension *grazing* of the Rep(eat)-Q also presented very good evidence in terms of internal consistency (ω*_L_*_1_ = 0.86; ω*_L_*_2_ = 0.90; ω*_partial L_*_1_ = 0.95).

### 3.4. Testing the Proposed Structural Model

**Figure 2:**
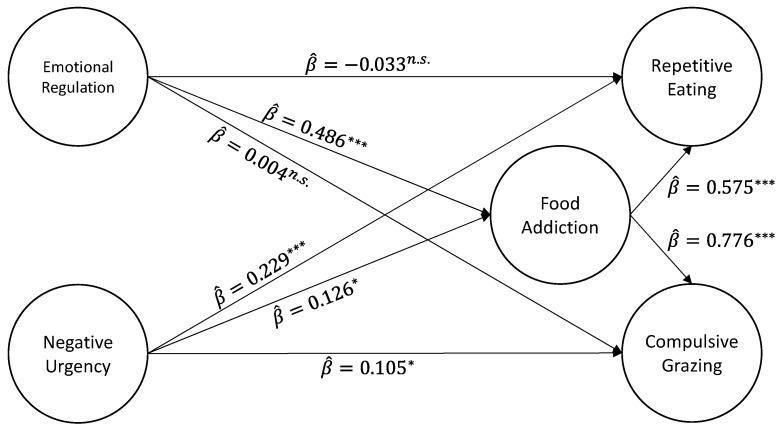
Structural equation model that describes the direct effects between variables of emotional regulation difficulties and negative urgency, food addiction symptomatology, and grazing behaviors (both subscales: Repetitive eating and Compulsive grazing). *—*p* ≤ 0.05. ***—*p* ≤ 0.001; *n*.*s*.—Not statistically significant.

The proposed structural model (Figure 2) presented an acceptable fit to the data (χ^2^_(1695)_ = 3167.575; *p* < 0.001; *CFI* = 0.955; *NFI* = 0.908; *TLI* = 0.953; *SRMR* = 0.085; *RMSEA* = 0.051; 90% *CI* (0.048; 0.053); *P*[*RMSEA* ≤ 0.05] = 0.318). The model presented satisfactory values of explained variance, namely 31.6% of the variability of food addiction scores (*r^2^_FA_* = 0.316), 67.9% of the variability of compulsive grazing scores (*r^2^_CG_* = 0.679), and 45.6% of the variability of repetitive eating (*r^2^_RE_* = 0.456).

Table 2 presents the tested (direct, indirect, and total) effects. All direct, indirect, and total effects were statistically significant, except for the direct effect of emotional regulation on compulsive grazing ($\hat{\beta }$ = 0.065; *p* = 0.233) and the direct effect of emotional regulation on repetitive eating ($\hat{\beta }$ = 0.035; *p* = 0.540). Food addiction partially mediated the effects of emotional regulation on compulsive grazing ($\hat{\beta }$ = 0.377; *p* < 0.001) and repetitive eating ($\hat{\beta }$ = 0.279; *p* < 0.001) and the effects of negative urgency on compulsive grazing ($\hat{\beta }$ = 0.098; *p* = 0.020) and repetitive eating ($\hat{\beta }$ = 0.072; *p* = 0.023).

## 4. Discussion

This study aimed to investigate the frequency of food addiction in a sample of university students and its role in understanding the association between negative urgency, difficulties in emotional regulation, and grazing behavior.

In this study’s sample, 10.7% of the university students exhibited symptoms and significant impairment consistent with a diagnosis of food addiction, and 54.4% of scores were within the clinical level of grazing. The presence of food addiction and grazing among the participants aligns with previously reported rates of these problems in similar populations [10,18,46]. However, there are still divergent results depending on the studied population, with systematic reviews indicating a prevalence of food addiction ranging from 0% to 25.7% for non-clinical populations [7,47]. Regarding the severity of food addiction, 60.7% of the participants with food addiction met the criteria for a severe diagnosis of food addiction.

### 4.1. The Mediation Model

Our findings support the proposed model, suggesting that food addiction partially mediates the effect of difficulties in emotional regulation and negative urgency on grazing behavior (both subscales). This model explains a significant amount of variability of food addiction (31.6%), compulsive grazing (67.9%), and repetitive eating (45.6%). Other authors also suggested that both impulsivity and a lack of emotional regulation strategies facilitate the consumption of highly palatable foods [16].

These findings indicate that individuals with greater impulsivity traits and lower emotional regulation capacity have a higher risk of developing problematic eating behaviors, partially due to food addiction. In situations of intense and unpleasant emotions, individuals struggle to regulate their emotions, having a more immediate orientation and a greater tendency to act impulsively [27,28]. On such occasions, they are more likely to use food as a coping strategy to deal with their negative emotions, particularly when they score higher on food addiction, in order to decrease their states of negative affect. Accordingly, food addiction has been considered a dysfunctional emotional regulation coping strategy [23,48,49]. In line with this hypothesis, past research analyzing the associations between food addiction, personality traits, and negative affect regulation showed that women with a diagnosis of food addiction, compared to those without, used more externalizing behavioral strategies (e.g., eating) to soothe or cope with negative emotions [50]. Despite this direct effect on food addiction symptoms, our results indicate that difficulties in emotion regulation alone do not have a direct effect on grazing. The loss of control preceding food addiction, along with difficulties in emotional regulation, increases the likelihood of engaging in grazing behaviors [51].

Finally, our results showed that the associations between food addiction and grazing were stronger in the compulsive grazing scale than in the repetitive eating subscale. The additive mechanisms associated with food consumption present in food addiction may trigger the consumption of small portions of food—especially hyperpalatable foods—throughout the day in a repetitive and unplanned manner (grazing) [13,16]. This is consistent with previous studies that have found a strong association between compulsive grazing and the severity of food addiction symptoms, which considered compulsive grazing to be an addictive behavior in response to food [12,51]. These authors argue that the overlap between the characteristics of these variables, such as loss of control and excessive intake, are similar to patterns observed in substance disorders [12,51].

### 4.2. Limitations

It should be noted that there are some limitations to consider, such as the cross-sectional nature of this study, which does not allow for causal inferences regarding the relationship between variables. Additionally, the fact that the participants were mostly female limits the generalizability of the results to other genders. In the future, it would be advantageous to include a more balanced number of men and women in the sample and to increase the sample size for possible comparisons between groups with and without food addiction and grazing. Finally, the use of self-report instruments may also be a disadvantage, as these measures bias are known to overreport problematic eating.

## 5. Conclusions

Food addiction is an emerging concept; however, there is still a lack of understanding about the interplay between food addiction, grazing, and psychological variables among college students, who are a population at risk for developing problematic eating behaviors. Results supported the mediating role of food addiction, indicating that the association between difficulties in emotional regulation and negative urgency with grazing is partially explained by the psychopathology of food addiction. Furthermore, these results highlight the compulsive component of food addiction and its relationship with the inadequacy of resources to regulate emotions adaptively. This underscores the importance of assessing these variables, particularly in populations at risk for the development of disordered eating problems and eating disorders, such as university students. Efforts to prevent the development of problematic eating behaviors should consider the design of interventions focused on emotion regulation and impulse control.

## Figures and Tables

**Table 1:** The correlational matrix between the symptomatology of food addiction with difficulties of emotional regulation, negative urgency, and grazing (Compulsive and Repetitive Eating).

Variables	*M*	*SD*	1	2	3	4	5	6
1. Symptomatology of Food Addiction	1.29	2.40	-----					
2. DERS-SF: Total	39.84	12.65	0.420 ***	-----				
3. UPPS–UN subscale	26.67	7.38	0.411 ***	0.613 ***	----			
4. Rep(Eat)-Q: Total	1.82	1.47	0.666 ***	0.442 ***	0.503 ***	-----		
5. Rep(Eat)-Q: CG	1.44	1.34	0.675 ***	0.443 ***	0.476 ***	0.928 ***	----	
6. Rep(Eat)-Q: RE	1.59	1.30	0.556 ***	0.374 ***	0.457 ***	0.924 ***	0.716 ***	----

Note. *n* = 338; *M*—mean; *SD*—standard deviation; DERS-SF: Total—total score the Difficulties in Emotion Regulation Scale–Short Form; UPPS-UN subscale—total score the negative urgency subscale of the Impulsive Behavior Scale; Rep(Eat)-Q: Total—total score the Repetitive Eating Questionnaire; Rep(Eat)-Q: CG—compulsive grazing subscale of the Repetitive Eating Questionnaire; Rep(Eat)-Q: RE—repetitive eating subscale of the Repetitive Eating Questionnaire; ***—*p* < 0.001.

**Table 2:** Path Analysis: Direct, Indirect, and Total Effects.

Path	*B*	*se*	$\hat{\beta }$	*z*	*p*	95% *CI*
Direct Effects (Y ⇐ X)						
FA <- ER	0.489	0.061	0.486	8.084	<0.001	(0.371; 0.608)
FA <- NU	0.099	0.042	0.126	2.336	0.019	(0.016; 0.181)
CG <- FA	0.984	0.062	0.776	15.934	<0.001	(0.863; 1.105)
CG <- ER	0.005	0.071	0.004	0.077	0.939	(−0.134; 0.145)
CG <- NU	0.105	0.041	0.105	2.531	0.011	(0.024; 0.186)
RE <- FA	0.718	0.059	0.575	12.200	<0.001	(0.603; 0.834)
RE <- ER	−0.042	0.075	−0.033	−0.560	0.576	(−0.190; 0.105)
RE <- NU	0.225	0.047	0.229	4.772	<0.001	(0.132; 0.317)
Indirect Effects (Y ⇐ M ⇐ X)						
CG <- FA <- ER	0.482	0.067	0.377	7.196	<0.001	(0.350; 0.613)
RE <- FA <- ER	0.351	0.054	0.279	6.537	<0.001	(0.246; 0.457)
CG <- FA <- NU	0.097	0.042	0.098	2.324	0.020	(0.015; 0.179)
RE <- FA <- NU	0.071	0.031	0.072	2.279	0.023	(0.010; 0.132)
Total Effects (Y ⇐ X + [Y ⇐ M ⇐ X])						
CG <- ER + (CG <- FA <- ER)	0.487	0.080	0.381	6.067	<0.001	(0.330; 0.644)
RE <- ER + (RE <- FA <- ER)	0.309	0.076	0.246	4.092	<0.001	(0.161; 0.457)
CG <- NU + (CG <- FA <- NU)	0.202	0.059	0.203	3.395	0.001	(0.085; 0.318)
RE <- NU + (RE <- FA <- NU)	0.295	0.056	0.302	5.319	<0.001	(0.187; 0.404)

Note: ER—Emotional Regulation; FA—Food Addiction; CG—Compulsive Grazing; RE—Repetitive Eating; NU—Negative Urgency.

## Data Availability

The datasets analyzed during the current study are available from the corresponding author upon reasonable request.
